# Response surface methodological (RSM) approach for optimizing the removal of trihalomethanes (THMs) and its precursor’s by surfactant modified magnetic nanoadsorbents (sMNP) - An endeavor to diminish probable cancer risk

**DOI:** 10.1038/s41598-019-54902-8

**Published:** 2019-12-04

**Authors:** Minashree Kumari, Sunil Kumar Gupta

**Affiliations:** Department of Environmental Science and Engineering, Indian Institute of Technology (ISM), Dhanbad, 826004 Jharkhand India

**Keywords:** Environmental monitoring, Environmental impact

## Abstract

Response surface methodology (RSM) approach was used for optimization of the process parameters and identifying the optimal conditions for the removal of both trihalomethanes (THMs) and natural organic matter (NOM) in drinking water supplies. Co-precipitation process was employed for the synthesis of magnetic nano-adsorbent (sMNP), and were characterized by field emission scanning electron microscopy (SEM), trans-emission electron microscopy (TEM), BET (Brunauer-Emmett-Teller), energy dispersive X-ray (EDX) and zeta potential. Box-Behnken experimental design combined with response surface and optimization was used to predict THM and NOM in drinking water supplies. Variables were concentration of sMNP (0.1 g to 5 g), pH (4–10) and reaction time (5 min to 90 min). Statistical analysis of variance (ANOVA) was carried out to identify the adequacy of the developed model, and revealed good agreement between the experimental data and proposed model. The experimentally derived RSM model was validated using *t-test* and a range of statistical parameters. The observed R^2^ value, adj. R^2^, pred. R^2^ and “F-values” indicates that the developed THM and NOM models are significant. Risk analysis study revealed that under the RSM optimized conditions, a marked reduction in the cancer risk of THMs was observed for both the groups studied. Therefore, the study observed that the developed process and models can be efficiently applied for the removal of both THM and NOM from drinking water supplies.

## Introduction

Long-term exposure of disinfection by-product (DBPs) especially trihalomethanes (THMs) in drinking water is potentially harmful and is a cause of major health concern. Since 20^th^ century, chlorination has been widely used as a disinfectant to protect water against microbial growth during water treatment process^1^. Over the years, increasing attention has been paid on human health risks of THMs in drinking water supplies. THMs consists of four compounds i.e. chloroform (CHCl_3_), bromodichloromethane (CHBrCl_2_), dibromochloromethane (CHBr_2_Cl) and bromoform (CHBr_3_). The sum of four THMs is referred to as total THMs (TTHMs). Literature studies revealed that prolonged exposure of THMs leads to increased risk of different types of cancer such as bladder, colon, rectum, blood, stomach and rectal^2–4^. Toxicological and epidemiological studies have reported a direct link between THMs and cancer risk^3,4^.

Dissolved organic matter (DOM), mainly humic acids (HAs) and fulvic acids (FAs) are the major precursors for the formation of THMs in drinking water. A number of water quality operational parameters like pH, temperature, residual chlorine concentration, natural organic matter (NOM) etc., influences the formation of THMs. Water treatment plants (WTPs) removes only a specified percentage of DOM which decreases the rate of THM formation potential (THMFP) during the water treatment process as well as in the distribution systems^5^. But studies indicated that THMs are hydrophobic in nature and low molecular weight compound which makes the removal process challenging by most physio-chemical processes^6^. Treatment technologies such as membrane filtrations and reverse osmosis are effective in removing THMs but high cost of membrane and pumping limits their application in developing countries^7^. Other process such as adsorption has been extensively applied for the removal of organic and inorganic pollutants in water systems^7,8^. Over the years, nano-adsorbents has received substantial consideration and emerged as a novel adsorbent due to their unique magnetic properties, large surface area, less mass transfer resistance and removal of contaminants^8–10^. Applications of different types of nanoparticles like carbon based nanoparticles, metal oxide based nanoparticles, titanium oxide based nanoparticles and nano zerovalent iron in water treatment have been reported in literature^11–15^. Various methods have been adopted for the synthesis of MNPs, of which co-precipitation is the easiest and most convenient method and forms MNPs with fine size distribution.

THMs are a cause of great concern in drinking WTPs due to their probable cancer and non-cancer risk on human health^16,17^. Number of studies all across the world has reported cancer and non-cancer risk of THMs for individual age groups, and for male and female as well^2,18–21^. Additionally, it is quite difficult to remove THMs once they are formed in drinking water during the disinfection of water and possess huge challenge for the concerned authority and to the general public as well. Thus, the development of treatment technologies and optimization of process variables through statistical analysis is required to reduce the concentration of NOM and THMs in drinking water.

Response surface methodology (RSM), based on design of experiments is a set of statistical and mathematical tool for designing experiments and optimizing the effect process variables^22–24^. RSM reduces the number of trials and recognizes the influence of process parameters on the removal process^24,25^. RSM has been successfully used for the optimization of process parameters like adsorbent dose, pH, metal ion concentration, reaction time etc., for bio-sorption of metals^25,26^ and dyes^27^. RSM finds wide scale application in drinking water treatment process such as electrochemical^24^ and advanced oxidation process^28^. As far as known, no such study on the optimization of process variables using RSM approach for the removal of THMs and its precursors in drinking water has been reported in literature. Therefore, the prime objective of the present study was to analyze the effect of process parameters and optimization of conditions using RSM for the removal of NOM and THMs in drinking water. The variables employed in the experimental design were concentration of sMNP, pH and reaction time. Box-Behnken experimental design was used to optimize the process variables for THMs and NOM. Lastly, a study was carried out to analyze the probable risk under the optimized conditions to determine the reduction in risk associated.

## Materials and Methods

### Chemicals and reagents

Ferrous chloride (FeCl_2,_ M.W: 126.75 g/mol), ferric chloride (FeCl_3._6H_2_O, M.W: 162.2 g/mol), ammonium hydrochloride (NH_4_Cl, M.W: 53.489 g/mol) and ethanol (CH_3_CH_2_OH, M.W: 46.06 g/mol) was purchased from Merck. Polyethylene glycol (PEG), and THMs standard (purity 99%) was procured from Sigma Aldrich, Germany. All the chemicals and reagents used in the experiments were of analytical grade. The solutions were prepared in deionized water, and pH was adjusted by using NaOH or HCl (1 N).

### Preparation of sMNP

Chemical precipitation method was used for synthesis of nano-adsorbents^29^, according to the following reaction as given in Eq. 1:

1 $$F{e}^{2+}+2F{e}^{3+}+8O{H}^{-}\to F{e}_{3}{O}_{4}+4{H}_{2}O$$

**Figure 1:**
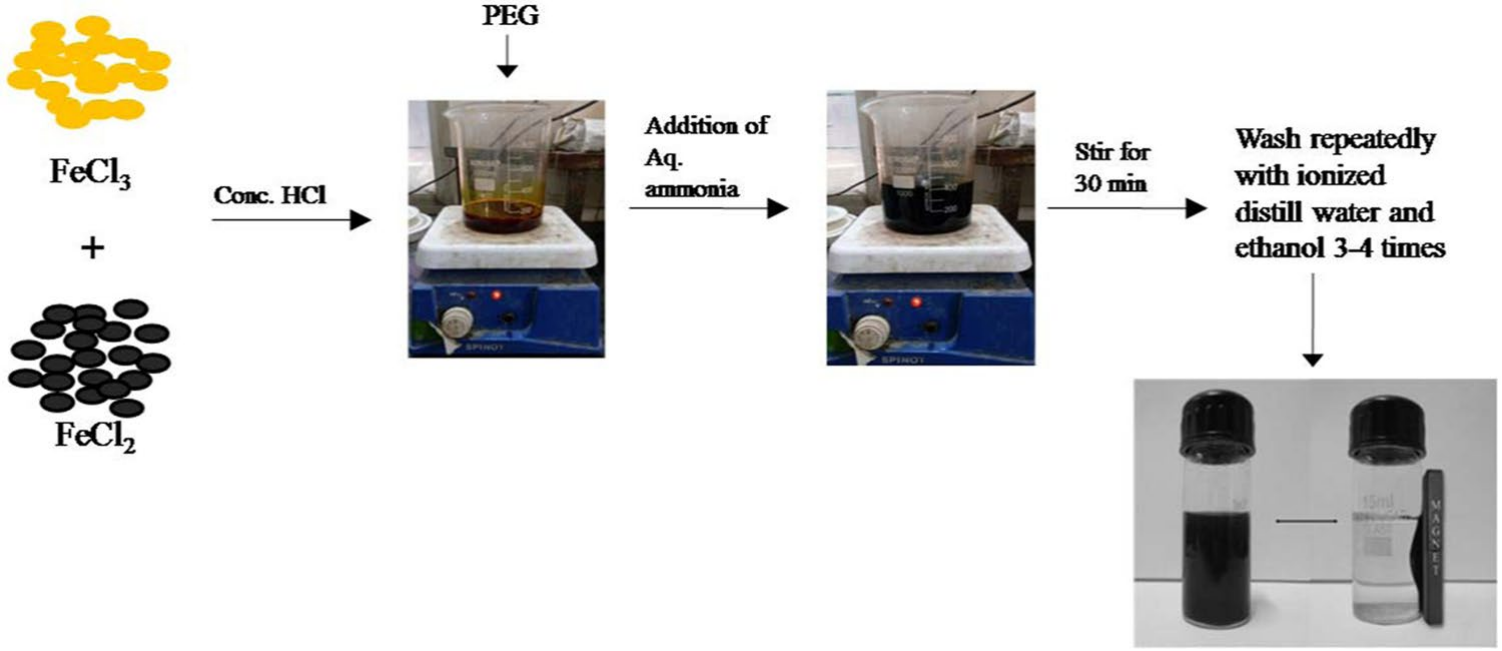
Schematic diagram for the synthesis of sMNP.

The method was optimized as per our needs and the detailed procedure is described as follows: (1) 1 M iron solution was prepared by adding ferrous and ferric chloride (2:1 ratio) in 100 mL of HCl with the addition of 0.25 g of PEG as surfactant. (2) 20 mL of aq. ammonia solution (30% w/w) as reducing agent was added drop wise (2 mL/min) to the solution under vigorous magnetic stirring. (3) The solution was shaken for another 30 min after addition of ammonia (4) The sMNP were rinsed with ethanol and deoxygenated distilled water (30:70 v/v). sMNP were then separated by a permanent magnet and dried in hot air oven at 60–70 °C to obtain black colored sMNP. Schematic representation for synthesis is shown in Fig. 1.

### Characterization of sMNP

The surface and morphological properties of sMNP was determined using a field emission scanning electron microscope (FE-SEM) (Model: Supra 55; Carl Zeiss, Germany) and ultra-high-resolution transmission electron microscope (TEM, model no. JEM 2100 HR; JOEL Ltd. Japan). BET (Brunauer–Emmett–Teller) surface area of sMNP was measured using a static nitrogen adsorption instrument at 77 K (Model JW-04 Beijing, China). Elemental composition of sMNP was analyzed by Energy Dispersive X-ray analysis (EDX) which was available as an attachment to FE-SEM. The electric charge present on the particles was measured as zeta potential (ζ) with the help of zeta potential analyzer (ZETA METER-4.0, USA). Phase identification analysis of sMNP was carried out by X-ray diffraction (XRD, Rigaku, Tokyo, Japan) whereas Fourier transform spectrometer (FT-IR) (Model Agilent Cary 660, US) was used for the determination of functional groups present in sMNP.

### Adsorption and degradation experiments

Batch adsorption experiments were carried out to study the effect of adsorbent dose, pH and contact time on THMs removal. Different dosages (0.1–3 g/L) of sMNP were taken in 100-mL conical flasks and desired concentration of THMs was added. The flasks were sealed properly and agitated at a speed of 125 rpm in a temperature-controlled water bath oscillator (Rivotek) until equilibrium was reached. pH of the solution (3.0–11.0) was adjusted by adding 0.1 N HCl or 0.1 N NaOH solutions, followed by pH measurement using pH meter (Model Phs-3C, Shanghai). Stock solution of THMs (1000 µg/mL) was procured from Sigma Aldrich. Adsorption capacity of sMNP, q_e_ (mg/g), was calculated using Eq. (2).

2 $${q}_{e}=\frac{({c}_{o}-{c}_{e})v}{m}$$

where, *C*_0_ and *C*_e_ are the initial and equilibrium concentration of NOM and THMs in the solution (mg L^−1^), m is the adsorbent dose (g), and V is the volume of the solution (L).

### Analytical methods

The qualitative and quantitative determination of TTHMs i.e. CHCl_3_, CHBrCl_2_, CHBr_2_Cl and CHBr_3_) were carried out using phase separation liquid-liquid extraction method. After extraction, the samples were transferred to 2 mL vials, and were analyzed by 800 CERES Plus Gas Chromatograph (GC) using Electron Capture Detector (ECD, Thermo-Fisher) equipped with Ni^63^catalyst. The detailed GC operating conditions is described in our previous work^30^. Total organic carbon (TOC) and dissolved organic carbon (DOC) content of the sample was analyzed by Shimadzu TOC analyzer (Model TOC-L/CSH/E200) as per the Standard Method 5910 B. DOC was analyzed after filtering the sample through 0.45 µm Millipore filter paper. THMFP was measured by Standard Method 5710-B^31^.

After the adsorption of NOM, remaining Fe content of sMNP was determined by hot plate digestion method. The samples were digested on an electric hot plate using a solution of HNO_3_, H_2_O_2_ and HF in 3:1:0.5 (v/v/v) for 2 hr. The resultant solution was dissolved in HNO_3_ and was diluted to the required volume with deionized water. The total Fe content of the particles was determined by Atomic absorption spectroscopy (AAS; Avanta, GBC, Australia). All the samples were analyzed in duplicate and the average values are reported.

### RSM experimental design

RSM is a multivariate statistical tool and offers a new approach to investigate the adsorption process. RSM provides better result reproducibility and process optimization with fine perspective for predictive model development. In RSM, response surfaces are graphical representation used to describe the interactive effects of process variables and their consequent effects on response^32,33^. Central composite design (CCD) and Box–Behnken design (BBD) are the two major factorial designs, used to assess the quadratic response surface and for developing second-order polynomial models in RSM^34,35^. CCD is a fractional factorial design while, BBD is a spherical, 3-level fractional factorial design, consisting of a central point and the middle points of edges of the circle circumscribed on the sphere^36^. The advantage of BBD is that it needs reasonably small group of parameters for determining the complex response function, and avoids experiments performed under severe conditions^37^. The present study used BBD for assessing the impact of process parameters on the response.

Process optimization involves estimation of coefficients, prediction of responses and checking acceptability of the developed model. The response is represented via Eq. (3):

3 $$Y=f({X}_{1},{X}_{2}\ldots \ldots \ldots \ldots \ldots \ldots {X}_{n})\pm E$$

where, *Y* is the response, *f* is the response function, X_1_…X_n_ are the independent variables, and E is the experimental error. The response function (*f*) largely depends on the nature of relationship between the response and the independent variables. The polynomial quadratic model is represented by Eq. (4):

4 $$Y={\beta }_{o}+{\sum }_{i=1}^{n}{\beta }_{i}{X}_{i}+{\sum }_{j=1}^{n}{\beta }_{ii}{X}_{i}^{2}+{\sum }_{i=1}^{n-1}{\sum }_{i}^{n}{\beta }_{ij}{X}_{i}{X}_{j}+E$$

where, *Y* is the predicted response; *β*_0_ represents the intercept or regression coefficient; *β*_*i*_*, β*_*ii*_ and *β*_*ij*_ represents the linear, quadratic and interaction coefficients; *X*_*i*_ and *X*_*j*_ are the coded values of the process variables; and E is the experimental/residual error.

In this study, BBD was developed using Design Expert (DX, 10.0.07; Stat Ease Inc., Minneapolis, USA) for optimizing three different independent process variables i.e. sMNP concentration, pH and reaction time on THMs and NOM removal, the dependent factors. Sequential experiments were carried out to develop the process. Initially, experiments were designed for 0 to 60 min reaction time, 0.1 to 1 g/L of MNPs and pH (varying from 4 to 10). Each numerical factor was varied over 3 levels i.e. low, medium and high. For developing a quadratic model, experiments were performed for at least three levels of each factor and the levels were equally spaced. Table 1 shows the actual values of the factors and their corresponding coded levels.

**Table 1:** Actual values of the factors and their corresponding coded levels.

Variable	Coded values		
	−1 (low)	0 (medium)	1 (high)
MNPs (g/L)	0.1	0.5	1
pH	4	7	10
Reaction Time (min)	1	60.5	120
TOC	2	6	10

In RSM, optimization of process variables comprises of seven different steps^38^. The steps include (1) Selection of response (THMs/NOM Removal), (2) Selection of variables and assigning codes to them, (3) Development of experimental design for removal of contaminants i.e. THMs/NOM, (4) Regression analysis, (5) Formation of a quadratic polynomial i.e. response development, (6) Developing 2D contour plot or 3D surface of the observed response surface, and at last (7) Analysis of optimum operating conditions.

### Validation of model

The mathematical model generated by RSM approach was validated by conducting experiment on given optimal medium setting and statistical *t*-test using various statistical parameters i.e., coefficient of determination (R^2^), adjusted R^2^ (R^2^adj) and predicted R^2^ (R^2^pred).

### Risk analysis

Cancer risk analysis was performed as per the USEPA protocol and detailed procedure is described elsewhere^20^. The risk was analyzed for all the three possible exposure routes i.e. oral ingestion, dermal absorption and inhalation. The value of input parameters used in risk estimation was adopted as per Indian conditions^2^. The obtained risk values were compared with those observed in Maithon WTP for male and female^21^ to determine the efficiency of optimized process variables for risk reduction.

### Quality control and precisions

All the samples were analyzed in triplicates to analyze the accuracy of measurements undertaken. For THM analysis, limit of detection was calculated for each individual THM by analyzing replicates of standard solution. The mean recovery THMs varied from 86.9% to 102.3%. Prior to sample analysis, GC was calibrated with a series of standards. The relative percentage difference (RPD) between two parallel samples was calculated and if the RPD exceeded ± 10%, the instrument was re-calibrated and samples were re-analyzed. The average of triplicate sample was considered as the final value.

## Result and Discussion

FE-SEM and zeta size analysis of sMNP indicates that the sMNP were less than 100 nm in size. sMNP size was further confirmed by TEM which specify the size to be 20 nm. BET analysis revealed that surface area of synthesized sMNP was 98.562 m^2^/g. Zeta potential analysis demonstrate negative charge on sMNP [(−) 58.35 mV to 74.9 mV, average value: 66.625 mV], and showed good to excellent stability against their mutual agglomeration. EDX analysis revealed that sMNP consists about 86.65% of iron whereas C, O and Au were present in 1.001%, 8.120% and 5.229%, respectively. The presence of Au was due to its use as a coating material during sample preparation for SEM-EDX analysis. The adsorption capacity, q_e_ of sMNP was found to be 59.92 mg/g (NOM removal) and 277.33 mg/g (THMs removal) depicting their excellent potential and capability towards both NOM and THMs removal. The detailed description and characterization of the synthesized sMNP was reported in previous study^29^.

### Optimization of experimental condition

**Figure 2:**
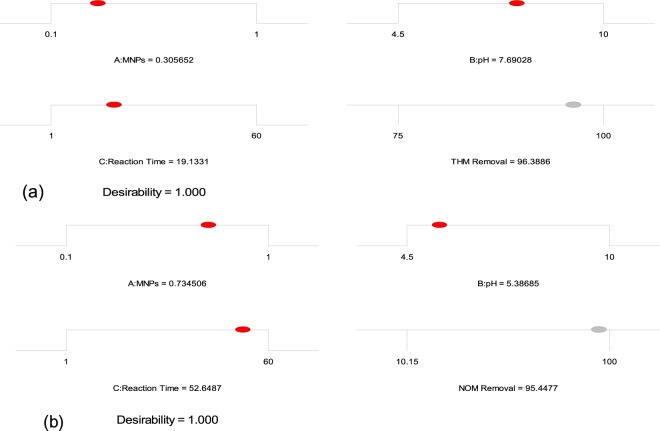
Numerical optimization of process variables (**a**) THM Removal (**b**) NOM Removal.

Experiments were conducted for the removal of both THMs and NOM from drinking water supplies using Design Expert model 9.0.10, USA. The upper limit of the process variables was wisely chosen for reducing chlorine dose, reaction time, and cost. The optimization program was used for setting the highest desirability and then different numerical combinations were looked for, maximizing the model functions. As shown in Fig. 2a, the optimized conditions of maximum responses for THMs removal was obtained at sMNP dose of 0.30 g/L, pH- 7.69 and reaction time of 19.13 minutes. Similarly, maximum NOM removal was achieved at sMNP dose of 0.734 g/L, pH of 5.38 and reaction time of 52.64 minutes (Fig. 2b). The optimized results were obtained at desirability (D) of 1.00, indicating the applicability of the developed models. D value closer to 1 is considered most desirable^38,39^. At optimized conditions, 96.38% THMs and 95.44% NOM removal was predicted by Eqs. 3 and 4, respectively. The obtained optimum conditions were further validated by an additional set of experiment to confirm the removal. This confirmatory run validated the accuracy of the model and reported 96.01% of THM and 95.21% NOM removal compared with those obtained using model equation. The experimentally observed response levels are in agreement with the model assumed theoretical values, indicating the precision and accuracy of the response surface models^40^.

### Development of response surface models

Response surface designs were tailored to fit the experimental results obtained from the design runs conducted in connection with the configured BBD. This resulted to an equation in coded forms, where, A, B, and C represented sMNP dose, pH and reaction time. Accordingly, the response, i.e. removal of THMs and NOM was computed using Eqs. 5 and 6, respectively.

- THM Removal

5 $$THM\,removal=+100.00+1.88A-1.25B+3.13C-(1.25AB)-(2.50AC)-(1.25BC)+{(0.0A)}^{2}-{(18.75B)}^{2}-{(2.50C)}^{2}$$

(b) NOM Removal

6 $$NOM\,removal=+100.00+10.17A+2.81B+25.55C+3.03AB-(10.35AC)-(3.59BC)-(7.42{A}^{2})-(15.77{B}^{2})-(27.16{C}^{2})$$

Analysis of variance (ANOVA) results for both models are given in Tables 2, 3. The developed model “F-value” was found to be 210.78 (THMs) and 12.18 (NOM), depicting that both these models are statistically significant. Large F values may occur due to noise and there is only 0.01% to 0.017% chance. Values of “Prob > F” were observed to be less than 0.0500, indicating that both the model terms are significant. Model terms A, B, C, AB, AC, BC, B^2^ and C^2^ are found to be significant for THMs model while in case of NOM removal, model terms A, C, B^2^ and C^2^ are. p values for both models were observed to be is <0.001, demonstrating the level of significance for developed models. If the p-values are greater than 0.10, the model terms are insignificant^29^.

**Table 2:** ANOVA of quadratic response surface model for THMs removal.

Source	Sum of	df	Mean	F	p-value	Remarks
	Squares		Square	Value	Prob > F	
Model	1693.75	9	188.19	210.78	<0.0001	Significant
A-MNPs	28.13	1	28.13	31.50	0.0008	
B-pH	12.50	1	12.50	14.00	0.0072	
C-Reaction Time	78.13	1	78.13	87.50	<0.0001	
AB	6.25	1	6.25	7.00	0.0331	
AC	25.00	1	25.00	28.00	0.0011	
BC	6.25	1	6.25	7.00	0.0331	
A^2^	0.000	1	0.000	0.000	1.0000	
B^2^	1480.26	1	1480.26	1657.89	<0.0001	
C^2^	26.32	1	26.32	29.47	0.0010	
Residual	6.25	7	0.89			
Lack of Fit	6.25	3	2.08			
Pure Error	0.000	4	0.000			
Cor. Total	1700.00	16

**Table 3:** ANOVA of quadratic response surface model for NOM removal.

Source	Sum of	df	Mean	F	p-value	Remarks
	Squares		Square	Value	Prob > F	
Model	11391.11	9	1265.68	12.18	0.0017	Significant
A-MNPs	827.43	1	827.43	7.96	0.0257	
B-pH	63.39	1	63.39	0.61	0.4603	
C-Reaction Time	5221.40	1	5221.40	50.25	0.0002	
AB	36.66	1	36.66	0.35	0.5712	
AC	428.28	1	428.28	4.12	0.0819	
BC	51.62	1	51.62	0.50	0.5037	
A^2^	231.74	1	231.74	2.23	0.1790	
B^2^	1047.63	1	1047.63	10.08	0.0156	
C^2^	3105.67	1	3105.67	29.89	0.0009	
Residual	727.36	7	103.91			
Lack of Fit	727.36	3	242.45			
Pure Error	0.000	4	0.000			
Cor. Total	12118.47	16				

**Figure 3:**
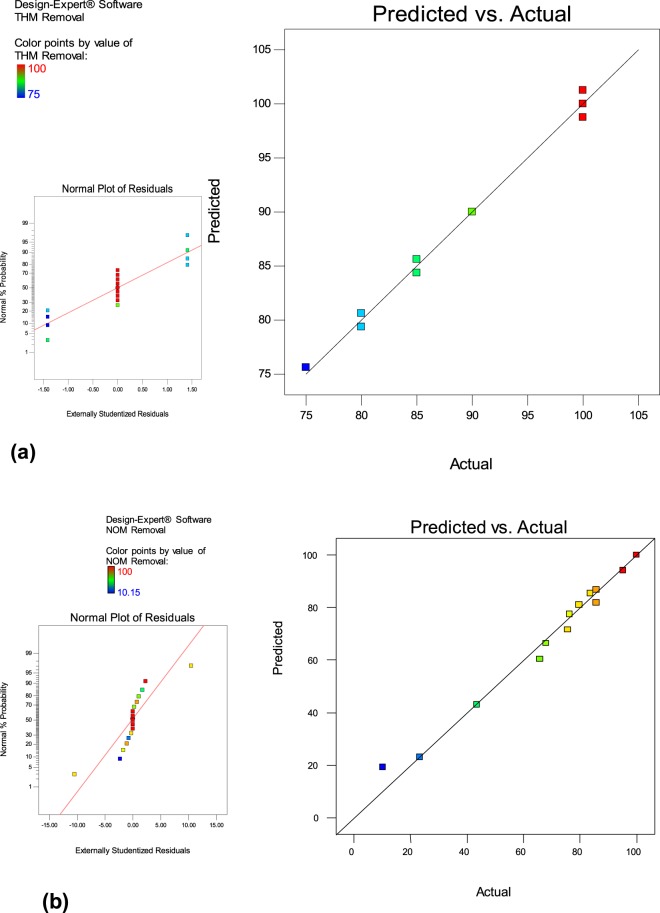
Graphical plot of predicted Vs actual values (**a**) THMs removal (**b**) NOM removal [Inside: Normal residual plots].

Besides p value, other statistical parameters such as coefficient of determination or R^2^, adjusted R^2^ (R^2^_adj_), predicted R^2^ (R^2^_pred_), coefficient of variation (CV%) etc., were also used to evaluate the competence of developed models^41^. R^2^ values and R^2^adj. for THMs removal was found to be 0.996 and 0.982. Similar values were obtained for NOM removal (R^2^ = 0.989 and R^2^adj. = 0.963) as well. The standard deviation for both the models were also found to be small i.e. 0.94 and 8.19 respectively. R^2^ values close to unity and smaller standard deviation values indicates better predicting response of the model developed^30,42^. The predicted R^2^ value was foundto be in close agreement with the adjusted R^2^. “Adeq Precision” determines the signal to noise ratio, of which a ratio more than 4 is desirable. In this study, the observed ratio for both models was found to be 99.42 and 5.92, respectively signifying the presence of adequate signal for navigating the design space. The developed model statistics is mentioned in Table 4. As shown in Fig. 3(a,b), the actual values are the measured response depicted by BBD and the predicted response is determined by using the approximate functions values for model evaluation. Straight line was obtained in normal % probability versus externally studentized residual plots for both these models (Fig. 3, inset Fig), thus showing normal distribution of data.

**Table 4:** Model statistics of the developed model.

Statistical parameters	Values of developed model	
	THMs removal	NOM removal
R^2^	0.996	0.989
Adjust R^2^	0.982	0.963
Pred. R^2^	0.971	0.939
Adeq. Precision	99.42	5.92
Std. dev.	0.94	8.19

### Model analysis via 2D contour graphs and 3-D surface plots

**Figure 4:**
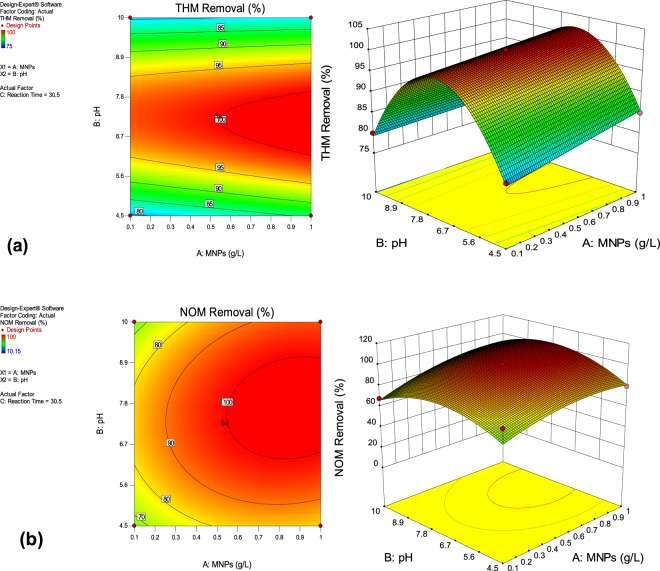
2D Contour and 3D surface plots (**a**) THMs removal (**b**) NOM removal.

To analyze the combined effect of the factors on THMs removal, graphical representation of the regression equation i.e. 2D contour graphs and 3-D surface plots were employed (Fig. 4a). The 2D contour plot represents that complete removal of THMs was obtained in reaction time of 30.5 min and at pH 7.2, whereas slightly higher pH 7.8 values were observed for NOM removal within same reaction time. But, when pH was increased to 10, marked decrease in the THMs and NOM removal efficiencies was witnessed as depicted from 3D surface plots (Fig. 4b).

### Cube plot

**Figure 5:**
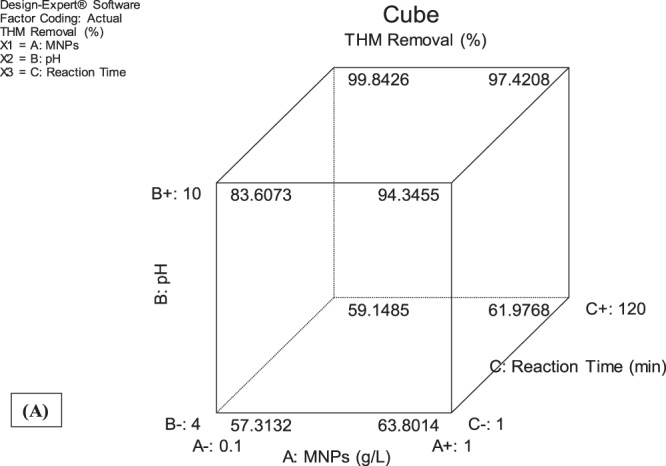
Cube plot (**A**) THM removal (**B**) NOM removal.

The outcome of the developed empirical models is shown via cube plot (Fig. 5A,B). The axis represents all the experimental design factors from low to high range whereas the coordinate point represents the outcome. The values depicted inside the cube represent the predicted removal efficiency of THMs by the process variable taken under study. Minimum THMs removal (57.31%) was achieved for low ranges of sMNP, pH and reaction time while maximum removal was achieved by high range of sMNP, reaction time and at neutral pH (99.84%) (Fig. 5A). Similar results were obtained for NOM removal, wherein minimum removal (45.03%) is obtained at low ranges of sMNP, pH and reaction time whereas maximum removal (94.56%) at high ranges (Fig. 5B).

RSM analysis revealed that the removal efficiencies decrease with increase in sMNP dose and pH. Complete removal of THMs and NOM was observed at 0.52 g/L and 0.56 g/L dose of sMNP. But, a decrease in removal efficiencies was reported at higher dose. This simultaneous decrease in THMs and NOM removal at high adsorbent dosage indicated that equilibrium of adsorption was reached and was found to be constant. Comparatively lower requirement of adsorbent dose found in our study may be attributed to finer size of sMNP i.e. 20 nm as evident from TEM analysis. The pH_pzc_ (point of zero charge) of MNPs was found to be 7.13 and at this pH, the total net surface charge on MNPs is zero. At low pH (<pH_pzc_), the MNPs surface has positive charge which attracts the negatively charged NOM molecules and thus enhances the rate of adsorption by reducing the zeta potential of sMNP via charge neutralization. Whereas at high pH (>pH_pzc_), the surface charge on the MNPs becomes negative which increases the electrostatic repulsion between NOM and MNPs surface, thereby reducing the NOM removal efficiency.

### Model validation

Experiments were performed under the predicted optimal conditions to verify optimization results. Design expert software predicted significant removal of THMs (99.84%) and NOM (94.56%) under optimized conditions. The experimental results closely agreed with those obtained using RSM and therefore validated the findings of response surface optimization. A *t*-test was also performed to determine the biasness of the developed models. *t*-test is a statistical tool for hypothesis testing and follows a student’s *t* distribution under the null hypothesis. *t*_*statistic*_ values for THMs (0.325) and NOM (0.296) were less than the *t*_*critical*_ value (2.01), and the p values were also greater than 0.05. This demonstrated that the model biasness is insignificant. A fairly good correlation was observed between the observed and predicted values (THMs: R^2^ 0.989; NOM: R^2^ 0.992), indicating that the developed models are statistically significant.

### Reduction in probable cancer risk

Due to the probable carcinogenic behavior of THMs on human health, it is imperative to develop treatment methods which will reduce their concentration in drinking WTPs. As reported in our previous work, the maximum concentration of TTHMs was found to be 511 µg/L in the treated water of Maithon WTP, Dhanbad, with an estimated cancer risk of 6.92 × 10^−4^ ^43^ wherein the risk assessment study was carried out in accordance with the USEPA protocol^44^. Under the RSM optimized conditions i.e. at an adsorbent dose of 0.30 g/L, pH 7.69 and reaction time of 19.13 min, the observed THMFP ranged from 36 to 42 µg/L. The risk analysis study revealed that under the optimized conditions, a marked reduction in the probable cancer risk was observed for all the three exposure routes in male and female (Table 5). Oral ingestion was the major route of exposure whereas risk due to dermal absorption and inhalation was found to be insignificant, as observed earlier. All the observed risk values were found to be less than 10^−6^, the acceptable risk level possessing no significant risk for any of the pathways studied. Thus, a marked reduction in the probable cancer risk of THMs on human health was observed under RSM optimized conditions.

**Table 5:** Risk reduction under optimized condition.

Route of exposure	Female		Male	
	At concentration observed in Maithon WTP	Under optimized condition (this study)	At concentration observed in Maithon WTP	Under optimized condition (this study)
Oral ingestion	8.08E-04	5.69E-06	6.92E-04	4.87E-06
Dermal Absorption	6.12E-09	4.31E-10	6.00E-09	4.22E-10
Inhalation	3.41E-07	2.40E-08	2.92E-07	2.06E-08

### Reuse of sMNP

Regeneration and reuse of the used or spent adsorbent (sMNP) is very critical in determining the applicability of the process applied. The study revealed that reusability of modified nano-adsorbents did not change significantly after five consecutive adsorption cycles for NOM (94.2–88.34%) and THMs removal (99.89–98.23%), respectively (Table 6). Out of the three desorbing solutions used, HCl has the maximum average desorbing efficiency of 96.72% and 97.59% respectively, for both NOM and THM removal. It was also observed that the increase in desorption cycle does not significantly alter desorption efficiencies caused due to the surface de-protonation of the adsorbent^45^.

**Table 6:** NOM and THMs adsorption and desorption % in 5 consecutive cycles of sMNP.

Cycle	% Adsorption		(Methanol)		% Desorption (HCl)		(De-ionized water)	
	NOM	THM	NOM	THM	NOM	THM	NOM	THM
1	94.2	99.89	67.6	73.12	98.8	99.99	17.12	10.78
2	93.8	99.56	64.5	71.67	97.6	98.12	16.15	8.67
3	91.56	99.12	61.2	69.32	96.5	97.67	15.98	7.56
4	90.12	98.99	60.13	67.21	95.9	96.23	13.67	5.89
5	88.34	98.23	58.5	66.15	95.1	95.96	9.15	5.06

In addition to this, the dissolved Fe concentration in the spent adsorbent was also determined to find out the stability of sMNP. AAS analysis revealed that the dissolved Fe concentration was found to be <0.2 mg/L in all the 5 cycles and hence, falls within the prescribed permissible limit for potable water i.e. 0.3 mg/L^46^. Accordingly, sMNP showed elevated operational reusability thereby diminishing the operational cost required for the synthesis of sMNP. Hence, sMNP exhibited ample amount of stability and is extremely capable in removing both NOM and THMs from drinking water supplies.

### Possible mechanism of THM degradation

Literature study reported that the iron particle helps in degrading carbon tetrachloride. Basically, Fe particles act as a reducing agent and provide electrons for the formation of H atoms which could possibly reduce the alkyl halides by hydrogenation^47^. Amongst THMs, brominated ones were easier to remove than chlorinated ones. This may be attributed to the difference in the bond dissociation energies (BDE) of carbon with chlorine and bromine atoms. BDE between the C-Cl atoms, (397 ± 29) kJ/mol, is more and stronger than that of between C-Br atoms (280 ± 21) kJ/mol^48,49^. This demonstrates that CHCl_3_ is likely to be the most stable compound, whereas CHBr_3_, the most unstable compound. Thus, the more stable molecule is less liable to undergo a chemical reaction because of the presence of strong bonds. Therefore, a strong relationship exists between THMs degradation and their respective BDE. In addition to this, bromine substituents are better leaving groups than chlorine substituent. Hence, the possible dehalogenation of THMs is in the order of: CHBr_3_ > CHBr_2_Cl > CHBrCl_2_ > CHCl_3_.

## Conclusions

sMNP exhibited excellent potential as an adsorbent under RSM optimized conditions for the removal of THM and its precursors from drinking water supplies. Mostly acidic pH is found effective for removal but this study revealed that complete removal of THM was obtained at pH close to neutral. Hence, suffices the problem of pH control in drinking water supplies. RSM seems to be a good tool for optimization of process variables. The experimentally determined response levels are in line with the model assumed theoretical values. Thus, confirming the accuracy and precision of the developed response surface models. Second order model best suitably described the response and was found to be appropriate in every experimental region. A marked reduction (>96%) in the probable risk was observed, thus, indicated the efficacy of sMNP for THM removal.
